# Impairment in Respiratory Function Contributes to Olfactory Impairment in Amyotrophic Lateral Sclerosis

**DOI:** 10.3389/fneur.2018.00079

**Published:** 2018-02-26

**Authors:** René Günther, Wiebke Schrempf, Antje Hähner, Thomas Hummel, Martin Wolz, Alexander Storch, Andreas Hermann

**Affiliations:** ^1^Division for Neurodegenerative Diseases, Department of Neurology, Technische Universität Dresden, Dresden, Germany; ^2^German Center for Neurodegenerative Diseases (DZNE) Dresden, Dresden, Germany; ^3^Smell & Taste Clinic, Department of Otorhinolaryngology, Technische Universität Dresden, Dresden, Germany; ^4^Department of Neurology, Elblandkliniken Meissen, Meissen, Germany; ^5^Department of Neurology, University of Rostock, Rostock, Germany; ^6^German Center for Neurodegenerative Diseases (DZNE) Rostock, Rostock, Germany

**Keywords:** amyotrophic lateral sclerosis, olfaction, hyposmia, sniffing, vital capacity, respiratory function

## Abstract

**Background:**

Nonmotor symptoms are very common in neurodegenerative diseases. In patients suffering from amyotrophic lateral sclerosis (ALS), olfactory dysfunction was first reported more than 20 years ago; however, its pathophysiological correlates and further implications remain elusive.

**Methods:**

In this so far largest case–control study, we analyzed olfactory performance with the “Sniffin’ Sticks,” a validated olfactory testing kit used in clinical routine. This test kit was designed to investigate different qualities of olfaction including odor threshold, odor discrimination, and odor identification.

**Results:**

ALS patients were mildly but significantly impaired in TDI score, the composite of the three subtests (ALS 27.7 ± 7.9, Controls 32.3 ± 5.8). In contrast to Parkinson’s disease, ALS patients did not show impaired performance in the suprathreshold tests identification and discrimination. However, the odor threshold was markedly decreased (ALS 6.0 ± 3.4, Controls 8.77 ± 3.6). This pattern of olfactory loss resembles sinonasal diseases, where olfactory dysfunction results from impeded odorant transmission to the olfactory cleft. The evaluation of medical history and clinical data of ALS patients showed that patients with perception of dyspnea (TDI 25.7 ± 8.0) performed significantly worse in olfactory testing compared to those who did not (TDI 30.0 ± 7.4). In line with that, we found that in patients with preserved respiratory function (vital capacity >70% of index value), olfactory performance did not differ from healthy controls.

**Conclusion:**

These findings suggest that the mild impairment of olfaction in patients suffering from ALS should at least partly be considered as a consequence of impaired respiratory function, and odor threshold might be a marker of respiratory dysfunction in ALS.

## Introduction

Amyotrophic lateral sclerosis (ALS) and its variants, primary lateral sclerosis and progressive muscular atrophy, are fatal neurodegenerative disorders with progressive degeneration of motor neurons and their axons (1–3). For many neurodegenerative diseases, it is well known that the pathology is not limited to the initially affected cell populations. Instead, disease spreading and involvement of other nonmotor regions in the brain seem to occur (4). Corresponding nonmotor symptoms like gastrointestinal, autonomic, neuropsychiatric, and sleep disorders are well known in these diseases. Treatments of such nonmotor symptoms (NMSs) are fundamental elements in a modern and comprehensive health care for patients suffering from neurodegenerative diseases. A wide range of NMS are known from Parkinson’s disease (PD) (5, 6). Frontotemporal impairment and memory loss are commonly known as “nonmotor” involvements in patients with ALS (7–10). Besides that, multiple other extra-motor symptoms in ALS have been reported in the last years (11, 12), for example, autonomic dysfunction (13–17), sensory (18), and extrapyramidal symptoms (19, 20). Neuroanatomical studies in sporadic forms of ALS revealed a disease stage-dependent distribution of phosphoTDP-43 in different, later also nonmotor, brain regions (3, 21, 22). In addition, substantia nigra hyperechogenicity, also considered as prodromal marker for PD, was found in ALS patients in similar frequency to PD (23).

We recently assessed the self-rating NMS questionnaire (NMSQuest) in patients suffering from ALS and its variants. Although the absolute frequency of NMS was not as high as in PD patients, ALS patients complained about impaired taste and/or smell significantly more often than healthy controls (5, 24). Hyposmia is one of the most common NMS in PD and can antedate classical motor symptoms by several years (25, 26). The olfactory system is one of the structures primarily affected in PD and is discussed as the starting point of disease propagation (27, 28). Olfaction impairment in ALS is not yet studied intensively. However, hyposmia was first reported in small populations of idiopathic ALS and later in Guamanian Chamorro ALS patients by means of the University of Pennsylvania Smell Identification Test (UPSIT) and cultural-adapted versions (29–32). A more comprehensive trial using the UPSIT in 58 ALS patients proved a slightly decreased overall smell identification compared to controls, but only patients with bulbar symptoms showed a significant impairment (33). More recently, a small prospective study (*N* = 26) using the 12 odors Sniffin Sticks test, a simple screening test, did not show any differences between ALS and matched controls with the limitation of a relatively small sample size (34). Another small study showed that hyposmia is common in a subgroup of ALS patients with cognitive impairment (35). Interestingly, a recent neuropathology study on postmortem samples of ALS patients showed that TDP-43-positive inclusion bodies could also be observed in the olfactory system (36).

The quality of olfactory function not only depends on the transmission and processing of odor information in the brain. Smelling is also dependent on various nonolfactory elements of the nose like airflow and mucociliar function. Respiration is one the main factors responsible for the active transport of stimulatory molecules to the sensory epithelia of the nasal cavity. The vast majority of olfactory dysfunction is caused by sinonasal diseases (37) including inflammatory diseases and respiratory dysfunction. Olfaction needs complex sensory-motor integration. Sniffing is the motor component which transports odorous substances to the olfactory receptors in the olfactory cleft, and olfactory performance depends on sniffing performance (38). Interestingly, hyposmia in PD is not exclusively caused by neurodegeneration; it is also partly caused by the impairment of sniffing, e.g., sniff airflow rate and sniff volume (39, 40). Sniff volume is one of the most important parameters for maximizing olfactory performance (41). Denervation of respiratory muscles and consecutive respiratory dysfunction with a decline of vital capacity is an obligatory phenomenon in ALS. Another sensitive parameter to predict respiratory dysfunction is the sniff nasal inspiratory pressure, and in a recent study, this parameter was shown to be more sensitive than vital capacity to predict the need for noninvasive ventilation. Sniff nasal inspiratory pressure declines very early and is much stronger than vital capacity in ALS (42).

The aim of this study was to screen for impairments in olfactory function, to assess the type of olfactory dysfunction, and to identify contributing factors in the so far largest case–control study concerning olfactory function in patients suffering from ALS.

## Materials and Methods

### Patients

Patients with definite, probable, or possible ALS, according to the revised El Escorial criteria (43, 44), as well as patients with primary lateral sclerosis and progressive muscular atrophy, were recruited from October 2011 to May 2017 at the Department of Neurology of the University Hospital Dresden, Technische Universität Dresden. Patients suffering from spinal muscular atrophy, spinal bulbar muscular atrophy (Kennedy syndrome), frontotemporal dementia overlap syndromes, and daily life affecting or clinically relevant dementia were not included. Patients with the stated diagnosis of pulmonary diseases or with diseases of the upper airway were not included; however, a diagnostic checkup for the presence of other pulmonary or upper airway diseases was not performed. In addition, an age- and sex-matched control group of healthy individuals was tested with the standardized “Sniffin’ Sticks” test kit. The study was approved by the institutional review board at the Technische Universität Dresden, and patients gave their informed consent.

### Assessments

Olfactory testing was performed using the “Sniffin’ Sticks” test kit (45, 46) which involves tests for odor threshold (“T”), odor discrimination (“D”), and odor identification (“I”). Odor identification was assessed for 16 common odors (orange, leather, cinnamon, menthol, banana, lemon, licorice, garlic, coffee, apple, pineapple, rose, fish, anise, clove, and turpentine) using a multiple forced choice design, namely subjects identify odors by selecting the best label from a list of four descriptors. Results of the three subtests are presented as a composite score (TDI) (“TDI score”) (range: 1–48), which is the sum of the results obtained for threshold (range: 1–16), discrimination (range: 0–16), and identification (range: 0–16) measures (45).

We additionally recorded age, gender, upper and lower motoneuron symptoms (UMNS, LMNS), bulbar symptoms, disease subtype at onset, forced vital capacity (FVC), FVC in percentage of predicted healthy index value (ppFVC)—which included adjustment for age, gender, and body height based on large databases of normative data (47, 48), body weight, body height, body mass index (BMI), disease duration (time between the first symptom and the date of test); the revised ALS-Functional-Rating Scale (ALSFRS-R); ALSFRS-R respiratory subscore, ALSFRS-R slope (the decline of ALSFRS-R from onset to the date of olfactory testing), complaining about dyspnea, tracheostomy, gastrostomy, noninvasive and invasive ventilation, REM sleep behavior disorder screening questionnaire (RBD-SQ), and NMSQuest.

### Statistical Analysis

As the samples were not normally distributed by calculation with Shapiro–Wilk test, statistical comparisons of data between groups were made using the non-parametric Mann–Whitney *U*-test (MWU) for comparisons of two groups and the non-parametric Kruskal–Wallis (KW)-ANOVA followed by median test dialog for multiple group comparisons. The chi-squared test (χ^2^) was carried out for a comparison of gender distribution between patients and controls. Spearman rank correlation coefficients were used to examine correlations between data of the “Sniffin’ Sticks” test kit and demographic as well as clinical data in the ALS group with a correlation coefficient of rho <0.3 considered as a weak, rho = 0.3–0.59 a moderate, and rho ≥0.6 a strong correlation. Data were analyzed using the software programs SPSS 21.0 (SPSS Inc., Chicago, IL, USA) and Statistica 13.2 [StatSoft (Europe) GmbH, Hamburg, Germany]. If not mentioned otherwise, all data are displayed as means ± standard deviation. Significance level was set at *p* < 0.05.

## Results

“Sniffin’ Sticks” test kit data from 94 MND patients and 81 healthy controls were analyzed and compared. Demographic and clinical characteristics of the study populations are shown in Table 1. The two groups did not differ significantly regarding age (MWU, *p* = 0.20) or sex (χ^2^, *p* = 0.06).

**Table 1:** Demographic and clinical characteristics of study populations.

	Control group	ALS group
Total number	81	94
Ratio of female in %	64.2	50.0
Age in years	62.7 ± 15.9	64.5 ± 10.2
Disease duration in years	–	2.4 ± 2.4
ALSFRS-R (range) Respiratory subscore	–	35.2 (18–48) 10.7 (4–12)
Subtype at onset (*n*)	–	Spinal (64)Bulbar (21)Primary lateral sclerosis (9)
UMNS in %	0	78.7
LMNS in %	0	90.4
Bulbar symptoms in %	0	52.7
Tracheostomy (*n*)	0	0
Gastrostomy (*n*)	0	3
Invasive ventilation (*n*)	0	0
Noninvasive ventilation (*n*)	0	7
Dyspnea in %	0	54.9
ppFVC in %	–	60.1 ± 27.5
FVC in ml	–	1984.1 ± 973.9
BMI	–	26.3 ± 4.5

### Mild Impairment of Olfactory Function in Patients Suffering from ALS

**Figure 1:**
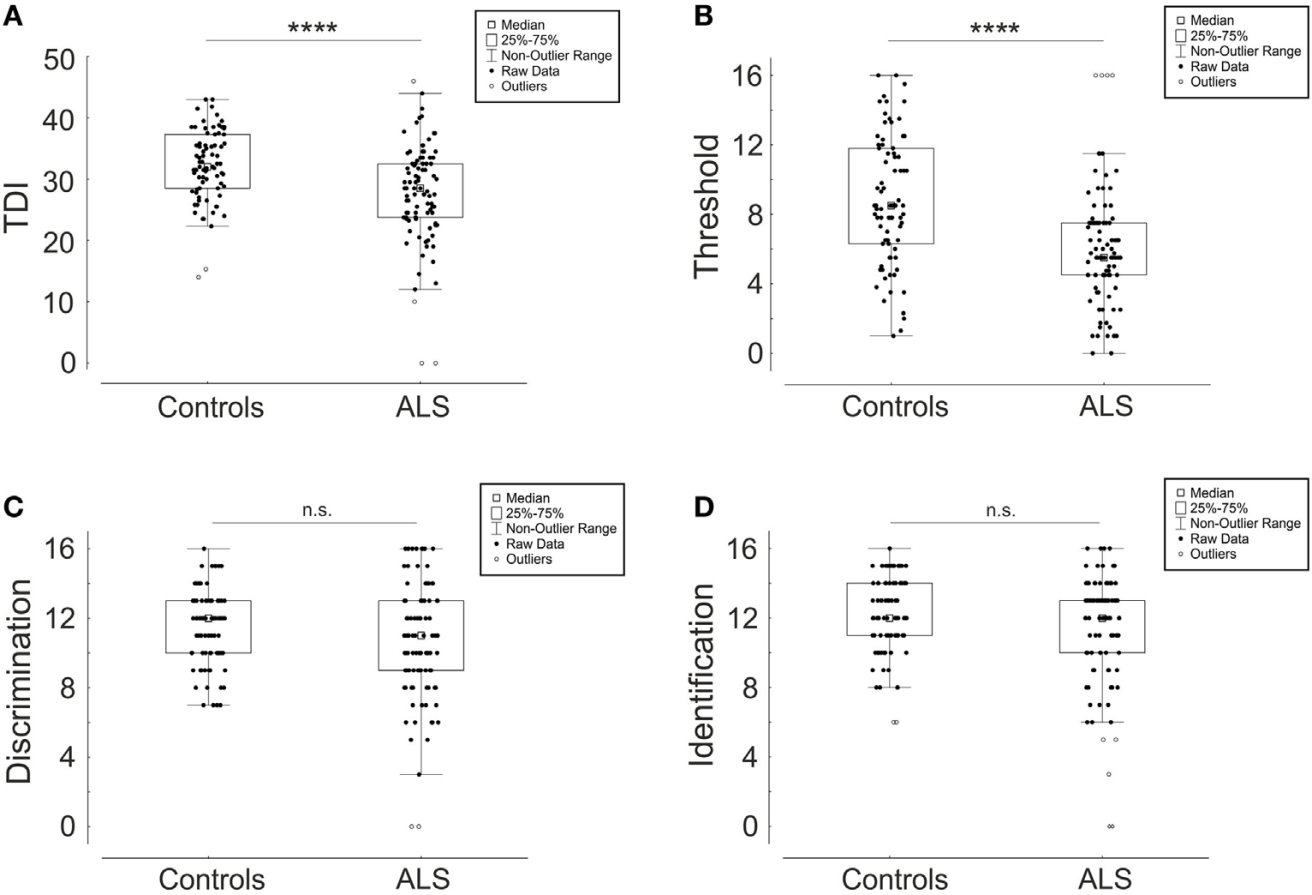
Comparison of “Sniffin Sticks” test results between amyotrophic lateral sclerosis (ALS) patients and healthy controls depicted as box plots with single values (dots). TDI score **(A)** is a composite of the three subtests threshold “T” **(B)**, discrimination “D” **(C)**, and identification “I” **(D)**. ****p < 0.0001, not significant (n.s.).

Patients suffering from ALS performed worse in comparison to healthy controls regarding total score “TDI” (ALS 27.7 ± 7.9, Controls 32.3 ± 5.8; *p* < 0.0001) and threshold “T” (ALS 6.0 ± 3.4, controls 8.77 ± 3.6; *p* < 0.0001) (Figures 1A,B). This means a loss of approximately 24% in “TDI” and 32% in “T”. Discrimination score “D” (ALS 10.6 ± 3.3, Controls 11.5 ± 2.1; *p* = 0.06) and identification score “I” (ALS 11.3 ± 3.2, Controls 12.0 ± 2.2; *p* = 0.32) were not significantly different to controls (Figures 1C,D). As demonstrated before on a large study group, people older than 55 years do not have sex-dependent differences in “TDI” score (46). Our study group consisted of people with a mean age over 60 years (Table 1), and we also did not find significant sex-dependent differences (TDI control group: males 32.9 ± 6.0, females 32.0 ± 5.7; *p* = 0.41; TDI ALS group: males 28.4 ± 7.6, females 27.1 ± 8.2; *p* = 0.26; also no differences between T, I, and D data shown).

### Motoneuron (MN) Disease Subtype Did Not Show Particular Impairment of Olfactory Performance

**Figure 2:**
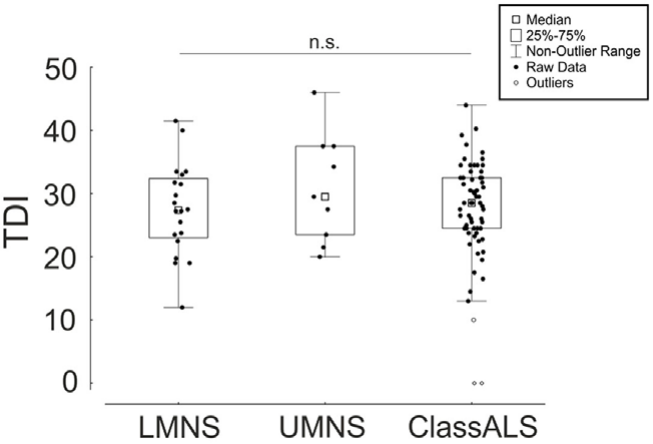
Comparison of the composite of the three subtests (TDI) between amyotrophic lateral sclerosis (ALS) patients with prominent lower motoneuron symptoms (LMNS), upper motoneuron symptoms (UMNS), and classical ALS with both symptoms (ClassALS). Box plots with single values (dots). Not significant (n.s.).

To find further influencing clinical parameters, we compared subgroups defined to clinically affected MN type at the date of test, labeled as only LMNS, only UMNS, and the classical ALS with affection of both MNs [classical ALS with both UMNS and LMNS (ClassALS)]. The three groups did not differ significantly for “TDI,” “T,” “D,” “I” (“TDI”: LMNS 27.5 ± 7.0, UMNS 30.8 ± 8.2, ClassALS 27.4 ± 8.1, *p* = 0.65; T, D, and I data not shown) (Figure 2).

### Mild Impairment of Olfactory Performance Occurs in Patients without Bulbar Symptoms (NoBulb)

**Figure 3:**
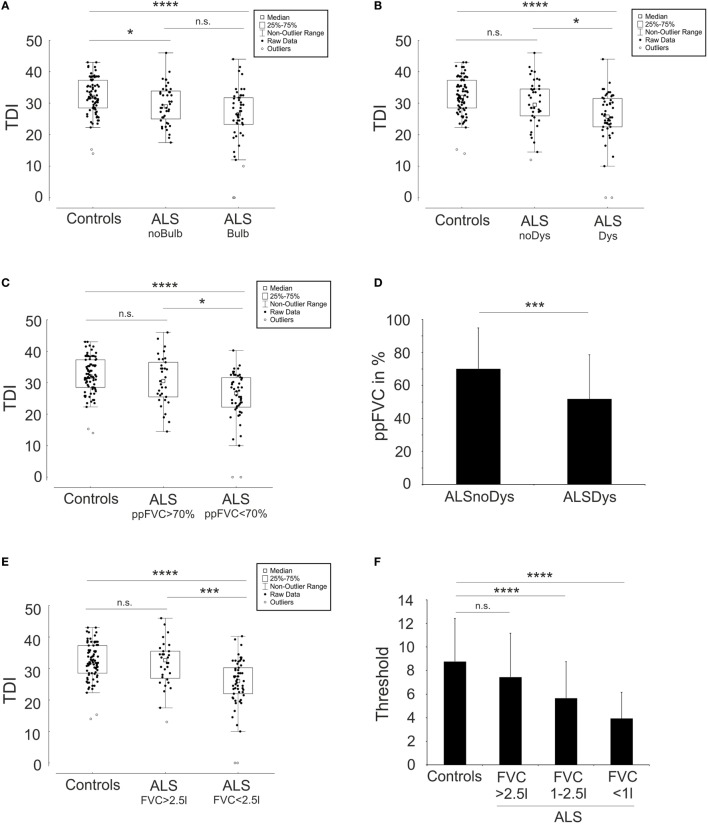
Olfactory dysfunction is a consequence of respiratory dysfunction. Depicted are box plots with single values (dots) concerning the comparison of the composite of the three subtests (TDI) scores of the “Sniffin Sticks” test results between amyotrophic lateral sclerosis (ALS) patients with (Bulb) and without (noBulb) bulbar symptoms **(A)**, the comparison between ALS patients with (Dys) and without (noDys) dyspnea **(B)**, the comparison between ALS patients with >70% and <70% forced vital capacity of predicted (ppFVC) **(C)**, and the comparison between ALS patients with >2.5l and <2.5l forced vital capacity (FVC) **(E)**. **(D)** shows the difference of ppFVC between ALS patients with (ALSDys) and without (ALSnoDys) dyspnea. In **(F)**, the decline of the subtest threshold “T” of the “Sniffin Sticks” test is depicted in comparison to FVC. ****p < 0.0001, ***p < 0.001, **p < 0.01, *p < 0.05, not significant (n.s.).

Previous published work hypothesized that hyposmia in patients suffering from ALS is only present in patients with bulbar symptoms (Bulb) (33). However, in our analysis, alterations occur in NoBulb for “TDI” (NoBulb 29.6 ± 6.2, Bulb 26.0 ± 9.1, Controls 32.3 ± 5.8, *p* < 0.00001 for Bulb vs Controls and *p* < 0.05 for NoBulb vs Controls) and “T” (NoBulb 6.4 ± 3.5, Bulb 5.6 ± 3.3, Controls 8.77 ± 3.6, *p* < 0.00001 for Bulb vs Controls and *p* < 0.001 for NoBulb vs Controls). “D” (NoBulb 11.2 ± 2.6, Bulb 10.0 ± 3.8, Controls 11.5 ± 2.1, *p* = 0.07) and “I” (NoBulb 12.3 ± 2.2, Bulb 10.5 ± 3.7, Controls 12.0 ± 2.2, *p* = 0.08 for Bulb vs Controls and *p* = 1.0 for NoBulb vs Controls) were not altered in both patients with and without bulbar symptoms. Although NoBulb and Bulb did not differ significantly in “TDI” and “T,” there is a propensity in the mean values toward worse performance in Bulb. In addition, a significant difference to less correct “I” (*p* < 0.05) in Bulb compared to that in NoBulb was calculated. But focused on statistical comparison to Controls, there is still not a relevant reduction of “I” performance in Bulb (see above). Taken together, bulbar palsy alone did not completely explain the worse Sniffin Sticks test results in patients suffering from ALS (Figure 3A).

### Patients Complained about Dyspnea Had Worse Olfactory Function

Olfactory impairment was evident in patients suffering from ALS, but in our results, the pattern of olfactory loss resembles sinonasal diseases, where olfactory dysfunction results from impeded odorant transmission to the olfactory cleft rather than a pattern of a neurodegenerative disease etiology like PD (49). Thus, we hypothesized that the obligate decline in the respiratory function of ALS patients is a contributing factor for the impairment in olfactory function. Therefore, we investigated the natural history of patients and compared patients who complained about dyspnea (ALSDys, *N* = 50) to patients who did not [ALS patients without dyspnea (ALSnoDys), *N* = 41]. Complaining of dyspnea was either recorded from the patients’ history and/or from ALSFRS-R respiratory subscore. “TDI” of ALSnoDys assimilates to “TDI” of control group and statistically changed into not significantly different. By contrast, “TDI” of ALSDys group performed worse than the baseline value of the overall ALS cohort and was significantly impaired in comparison to Controls and to ALSnoDys (ALSnoDys 30.0 ± 7.4; ALSDys 25.7 ± 8.0; Controls 32.3 ± 5.8; *p* < 0.00001 for ALSDys vs Controls and *p* = 0.26 for ALSnoDys vs Controls and p < 0.05 for ALSDys vs ALSnoDys) (Figure 3B). Although the total “TDI” was highly impaired in ALSDys group, “I” performance was not different in comparison to controls (ALSDys 10.8 ± 3.5; Controls 12.0 ± 2.2; *p* = 0.18). ALSDys group showed a slight reduction of “D” performance compared to controls (ALSDys 9.9 ± 3.6; Controls 11.5 ± 2.1; *p* < 0.05) and a strong reduction in “T” (ALSDys 5.2 ± 3.3; Controls 8.77 ± 3.6; *p* < 0.00001). ALSnoDys still performed worse in “T” compared to controls but better than the total ALS patient group and only slightly failed significance level to ALSDys group (ALSnoDys 6.9 ± 3.3; ALSDys 5.2 ± 3.3; Controls 8.77 ± 3.6; for ALSnoDys vs Controls; *p* < 0.05 and for ALSnoDys vs ALSDys; *p* = 0.06). Of note, patients in the ALSnoDys group still had a reduced mean ppFVC of 70%, and patients in the ALSDys group already had relevant involvement of respiratory function with a mean ppFVC of 50% (Figure 3D). Dyspnea was a subjective parameter and might not represent objective respiratory function.

### Respiratory Function Correlates with Impairment of Olfactory Function

To further evaluate clinical parameters that may correlate with the impairment of olfactory function, we performed several Spearman rank correlations (Table 2). There is a moderate correlation to age as already known from healthy people (46). Additionally weak to moderate correlations to weight and BMI were found for all “Sniffin Sticks” test parameters excluding “T.” No correlations were found to ALSFRS-R slope, NMSQuest, RBD-SQ, height, and disease duration. However, we found a highly significant correlation to FVC for all olfactory test parameters. Correlation to ppFVC showed a moderate correlation to “T” and a weak correlation to “TDI.” As expected, “D” and “I” did not correlate to ppFVC and were not altered in comparison to controls anyway. Although ppFVC was correlated moderately to ALSFRS-R (rho = 0.54, *p* < 0.00001) even when excluding the respiratory part (rho = 0.49, *p* < 0.00001), we found no correlation of “TDI,” “I,” or “D” to ALSFRS-R. However, there was a weak correlation of “T” to ALSFRS-R even when excluding the respiratory part. Taken together, we suggest that the respiratory function, probably the ability to sniff, was a prominent causal factor of the anyhow-weak impairment of olfactory function in ALS patients.

**Table 2:** Correlations of demographic and clinical characteristics to “Sniffin Sticks” test results in the ALS study group.

	TDI score	Threshold	Discrimination	Identification
Age	−0.59; *N* = 94****	−0.30; *N* = 94**	−0.42; *N* = 94****	−0.63; *N* = 93****
Height	n.s.; *N* = 91	n.s.; *N* = 91	n.s.; *N* = 91	n.s.; *N* = 91
Weight	0.31; *N* = 93**	n.s.; *N* = 93	0.26; *N* = 93*	0.27; *N* = 93**
BMI	0.28; *N* = 90**	n.s.; *N* = 90	0.22; *N* = 90*	0.23; *N* = 90*
FVC	0.41; *N* = 89****	0.35; *N* = 89***	0.31; *N* = 89**	0.32; *N* = 89**
ppFVC	0.25; *N* = 86*	0.31; *N* = 86**	n.s.; *N* = 86	n.s.; *N* = 86
ALSFRS-R	n.s.; *N* = 85	0.32; *N* = 85**	n.s.; *N* = 85	n.s.; *N* = 85
Only resp subscore	0.27; *N* = 84*	0.27; *N* = 84*	n.s.; *N* = 84	n.s.; *N* = 84
Without resp subscore	n.s.; *N* = 84	0.28; *N* = 84**	n.s.; *N* = 84	n.s.; *N* = 84
ALSFRS-R slope	n.s.; *N* = 85	n.s.; *N* = 85	n.s.; *N* = 85	n.s.; *N* = 85
Disease duration	n.s.; *N* = 94	n.s.; *N* = 94	n.s.; *N* = 94	n.s.; *N* = 94
RBD-SQ	n.s.; *N* = 61	n.s.; *N* = 61	n.s.; *N* = 61	n.s.; *N* = 61
NMSQuest	n.s.; *N* = 64	n.s.; *N* = 64	n.s.; *N* = 64	n.s.; *N* = 64

****p < 0.0001, ***p < 0.001, **p < 0.01, *p < 0.05; not significant (n.s.).

### Decline of Respiratory Function Was Associated with Worse Olfactory Performance

To further prove the assumed association of olfactory decline with respiratory performance, we divided ALS patients into two groups of patients with FVC below (ALSppFVC <70, *N* = 52) and above 70% (ALSppFVC >70, *N* = 34) of their predicted healthy index value. Of note, ppFVC includes an adjustment for age and gender to exclude age- and gender-dependent influences. “TDI” in ALSppFVC >70 was similar to controls, but by contrast, it was significantly reduced in ALSppFVC <70 (ALSppFVC >70, 30.7 ± 7.66; ALSppFVC <70, 25.5 ± 8.0; Controls 32.3 ± 5.8; *p* = 0.62 for ALSppFVC >70 vs Controls, *p* < 0.00001 for ALSppFVC <70 vs Controls, *p* < 0.05 for ALSppFVC >70 vs ALSppFVC <70) (Figure 3C). The same results were found concerning the total FVC (Figures 3E,F). Although there was a highly significant reduction in “TDI” in the ALSppFVC <70 group compared to controls, “I” performance did not differ from controls (ALSppFVC <70, 10.7 ± 3.6; Controls 12.0 ± 2.2; *p* = 0.16). “D” and “T” performances in ALSppFVC <70 group were significantly worse in comparison to controls and to ALSppFVC >70 (“D”: ALSppFVC >70, 11.7 ± 3.1; ALSppFVC <70, 9.8 ± 3.4; Controls 11.5 ± 2.1; *p* < 0.05 for ALSppFVC <70 vs ALSppFVC >70 and *p* < 0.01 for ALSppFVC <70 vs Controls) (“T”: ALSppFVC >70, 7.15 ± 3.7; ALSppFVC <70, 5.2 ± 3.2; Controls 8.77 ± 3.6; *p* < 0.00001 for ALSppFVC <70 vs Controls and *p* < 0.05 for ALSppFVC <70% vs ALSppFVC >70%). Patients in ALSppFVC >70 did not significantly differ from the control group in either “TDI,” “T,” “D,” or “I” performance.

## Discussion and Conclusion

Olfactory dysfunction is very common in neurodegenerative diseases, and in PD, hyposmia can precede motor symptoms for years (25, 26). The significance of NMS in patients suffering from ALS and its variants is not fully elucidated yet, but scientific evidence is increasing (12). Aside from a handful of publications concerning olfactory function, we recently reported hints for olfactory dysfunction in patients suffering from ALS and its variants (24, 29–33). In the current study, we analyzed olfactory function in ALS patients with the “Sniffin’ Sticks” (45, 46), a testing device used in clinical routine. In line with previous publications, we found a mild but significant decrease of olfactory performance compared to an age- and sex-matched control group. It is not yet known whether olfactory impairment in ALS patients and its variants is a consequence of the disease spreading in the brain with neurodegeneration in the olfactory system. We did not find differences between patients with a prominent impairment of the lower MNs and those with a prominent impairment of the upper MNs. Therefore, we cannot conclude easily that the mild olfactory impairment is predominantly a result of the disease spreading from the upper MNs.

Previously published work hypothesized that only patients suffering from bulbar symptoms present with olfactory impairment maybe as a consequence of a nonolfactory mechanical problem caused by bulbar palsy (33), but in our study, a significant lowering of olfactory performance was also evident in NoBulb. Thus, the hypothesis that hyposmia is a special symptom of patients with bulbar palsy should be further evaluated. Nasal airway ventilation, especially sniffing performance, is an important prerequisite to maximize odor performance (38). Thus, odor performance depends on sniffing performance and especially on the sniff volume (38, 41). Disease progression of ALS and its variants ends in the denervation of the respiratory apparatus, and some patients show a decline of vital capacity already in early disease stages. A recently published work showed a continuous decline of FVC in ALS patients prior to indication for noninvasive ventilation and provided evidence that sniff nasal inspiratory pressure decline was even much stronger (42). This is in agreement with our study showing an association between olfactory impairment and respiratory decline. ALSnoDys or with FVC above 70% of their predicted healthy index value (ppFVC) performed similar to controls. By contrast, only patients with dyspnea or with ppFVC below 70% had a worse olfactory performance than controls. Although patients with ppFVC below 70% had a reduction in general olfactory function, no significant reduction of odor identification performance was found. In these patients, the most prominent impaired parameter was olfactory threshold. As threshold testing yields the concentration above which an odor is perceived, test concentrations are very low in comparison with discrimination and identification being suprathreshold tests (49). This fact suggests that in the latter tests, odor concentrations are still high enough in the olfactory cleft, also in the case of a bad sniffing performance. It might also suggest that the transmission and processing of odors is not altered to a relevant extent in patients suffering from ALS and its variants even if respiratory function is declined. ALS-FRS-R, a score for severity and progression of ALS disease, was associated with a decline of respiratory function. “TDI,” “D,” and “I” showed no correlation to ALS-FRS-R; however, there was a weak correlation of “T” to ALS-FRS-R score even when excluding the respiratory part.

Our results do not support an early, clinical relevant neurodegeneration of brain regions acting for the transmission and processing of odors in patients suffering from ALS and its variants. Neuropathological studies in sporadic forms of ALS hypothesize a disease spread starting from motor cortex to effector regions and if ever involve olfactory transmission and processing systems very late in disease progression (21). By contrast, disease spreading or transmission in PD is thought to start in nonmotor regions, especially in the olfactory system (27). In line with that, PD patients perform relatively well in odor threshold but poorly in odor discrimination and identification which mainly depends on the transmission and processing of odor information in the brain, thus hinting toward primary neuronal dysfunction/degeneration (49).

However, we cannot fully exclude that the clinically relevant reduction of olfactory performance due to neurodegeneration occurs in patients suffering from ALS and its variants in advanced disease stages in which the examination would be very difficult with conventional odor-testing methods. Further neuropathological and MRI-based studies of olfactory bulb and olfactory-processing regions may help to further study neurodegeneration in the olfactory brain system in patients of ALS and its variants. Also, prospective studies are warranted to further evaluate the association and impact of olfactory function with/on respiratory function in ALS patients.

In summary, the reduction of olfactory performance in ALS and its variants should be considered as a consequence of impairment of respiratory function, and olfactory threshold testing might be an early marker of respiratory decline in ALS. However, because of the case–control study design with its limitations, further prospective studies of odor threshold testing in combination with multiple cardiorespiratory parameters such as vital capacity, sniffing capacity, and diaphragm ultrasound are needed to estimate its significance and diagnostic potential in patients suffering from ALS and its variants.

## Ethics Statement

The study was approved by the institutional review board at the Technische Universität Dresden (EK 393122012, EK 49022016), and patients gave their informed consent.

## Author Contributions

RG: Conception and design, collection and assembly of data, data analysis and interpretation, manuscript drafting. WS, AH, TH, MW, and AS: Collection and/or assembly of data, critical revision of manuscript. AH: Conception and design, principal investigator, collection and assembly of data, data analysis and interpretation, manuscript drafting.

## Conflict of Interest Statement

The authors declare that the research was conducted in the absence of any commercial or financial relationships that could be construed as a potential conflict of interest.

## Abbreviations

ALS, amyotrophic lateral sclerosis; ALSDys, patients with dyspnea; ALSnoDys, patients without dyspnea; ALSppFVC <70, patients with FVC below 70% of their predicted healthy index value; ALSppFVC >70, patients with FVC above 70% of their predicted healthy index value; ALSFRS-R, revised ALS-Functional-Rating Scale; BMI, body mass index; MWU, Mann–Whitney *U*-Test; NMS, Nonmotor symptoms; Bulb, patients with bulbar symptoms; ClassALS, classical ALS with both UMNS and LMNS; D, odor discrimination; FVC, forced vital capacity; I, odor identification; PD, Parkinson’s disease; KW, Kruskal–Wallis-ANOVA followed by median test dialog; LMNS, lower motoneuron symptoms; MN, motoneuron; NoBulb, patients without bulbar symptoms; ppFVC, FVC in percentage of predicted healthy index value; T, odor threshold; TDI, composite of T, I, D; UMNS, upper motoneuron symptoms; UPSIT, University of Pennsylvania Smell Identification Test; χ^2^, chi-squared test.
